# 4-Carbamoylpiperidinium acetate monohydrate

**DOI:** 10.1107/S1600536810045538

**Published:** 2010-11-13

**Authors:** Graham Smith, Urs D. Wermuth

**Affiliations:** a Faculty of Science and Technology, Queensland University of Technology, GPO Box 2434, Brisbane, Queensland 4001, Australia

## Abstract

In the structure of the title compound, C_6_H_13_N_2_O^+^·C_2_H_3_O_2_^−^·H_2_O, the amide H atoms of the cations form centrosymmetric cyclic hydrogen-bonding associations incorporating two water mol­ecules [graph set *R*_4_^2^(8)], which are conjoint with cyclic water-bridged amide–amide associations [*R*_4_^4^(12)] and larger *R*_4_^4^(20) associations involving the water mol­ecule and the acetate anions, which bridge through the piperidinium H-bond donors, giving an overall three-dimensional framework structure.

## Related literature

For structural data on isonipecotamide salts, see: Smith *et al.* (2010 ▶); Smith & Wermuth (2010*a* ▶,*b* ▶). For graph-set motifs, see: Etter *et al.* (1990 ▶).
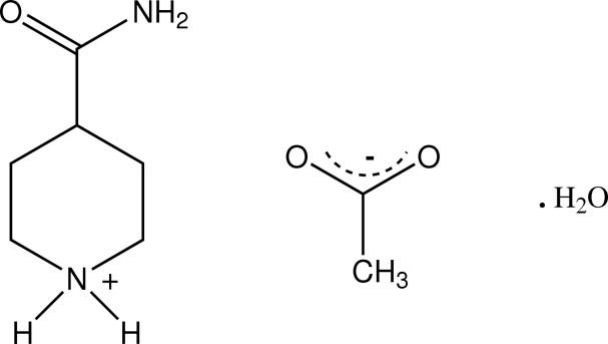


## Experimental

### 

#### Crystal data

C_6_H_13_N_2_O^+^·C_2_H_3_O_2_^−^·H_2_O
*M* *_r_* = 206.24
Triclinic, 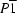

*a* = 5.8219 (2) Å
*b* = 7.7675 (3) Å
*c* = 12.4022 (5) Å
α = 81.088 (4)°
β = 78.763 (4)°
γ = 76.202 (4)°
*V* = 530.75 (4) Å^3^
*Z* = 2
Mo *K*α radiation
μ = 0.10 mm^−1^
*T* = 200 K
0.40 × 0.35 × 0.15 mm

#### Data collection

Oxford Diffraction Gemini-S Ultra CCD-detector diffractometer
Absorption correction: multi-scan (*CrysAlis PRO*; Oxford Diffraction, 2010 ▶) *T*_min_ = 0.962, *T*_max_ = 0.980
6385 measured reflections
2087 independent reflections
1602 reflections with *I* > 2σ(*I*)
*R*_int_ = 0.021

#### Refinement

*R*[*F* ^2^ > 2σ(*F* ^2^)] = 0.035
*wR*(*F* ^2^) = 0.105
*S* = 0.93
2087 reflections
151 parameters
H atoms treated by a mixture of independent and constrained refinement
Δρ_max_ = 0.20 e Å^−3^
Δρ_min_ = −0.19 e Å^−3^

### 

Data collection: *CrysAlis PRO* (Oxford Diffraction, 2010 ▶); cell refinement: *CrysAlis PRO*; data reduction: *CrysAlis PRO*; program(s) used to solve structure: *SIR92* (Altomare *et al.*, 1994 ▶); program(s) used to refine structure: *SHELXL97* (Sheldrick, 2008 ▶) within *WinGX* (Farrugia, 1999 ▶); molecular graphics: *PLATON* (Spek, 2009 ▶); software used to prepare material for publication: *PLATON*.

## Supplementary Material

Crystal structure: contains datablocks global, I. DOI: 10.1107/S1600536810045538/nk2071sup1.cif

Structure factors: contains datablocks I. DOI: 10.1107/S1600536810045538/nk2071Isup2.hkl

Additional supplementary materials:  crystallographic information; 3D view; checkCIF report

## Figures and Tables

**Table 1:** Hydrogen-bond geometry (Å, °)

*D*—H⋯*A*	*D*—H	H⋯*A*	*D*⋯*A*	*D*—H⋯*A*
N1*A*—H11*A*⋯O12^i^	0.940 (17)	1.793 (17)	2.7311 (16)	175.8 (17)
N1*A*—H12*A*⋯O11^ii^	0.949 (18)	1.824 (18)	2.7666 (16)	171.8 (14)
N41*A*—H41*A*⋯O1*W*	0.919 (18)	1.984 (18)	2.8939 (17)	170.2 (15)
N41*A*—H42*A*⋯O1*W*^iii^	0.899 (17)	2.188 (16)	2.9491 (16)	142.1 (15)
O1*W*—H11*W*⋯O11	0.92 (2)	1.87 (2)	2.7871 (15)	172 (2)
O1*W*—H12*W*⋯O41*A*^iv^	0.84 (2)	1.90 (2)	2.7370 (15)	177 (2)

Symmetry codes: (i) 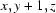; (ii) 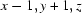; (iii) 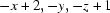; (iv) 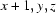.

**Fig. 1.**
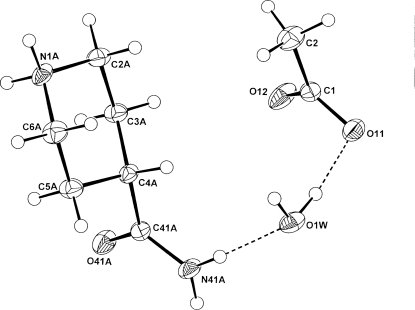
Molecular configuration and atom naming scheme for the three INIPA cation the acetate anion and the water molecule of solvation in (I). Inter-species hydrogen bonds are shown as dashed lines and displacement ellipsoids are drawn at the 40% probability level.

**Fig. 2.**
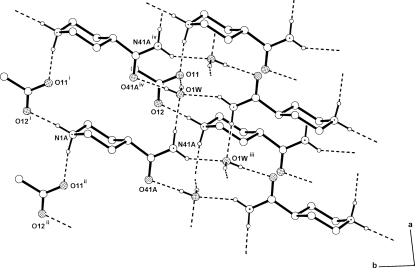
The three-dimensional hydrogen-bonded framework structure of (I) viewed down the approximate b cell direction showing the cyclic R24(8), R44(12) and R44(20) hydrogen-bonding interactions in (I). Non-associative H atoms are omitted. For symmetry codes, see Table 1.

## supplementary crystallographic information

### Comment

The amide 4-carbamoylpiperidine (isonipecotamide, INIPA) has proved to be a particularly useful synthon for the construction of crystalline salts with a range of aromatic carboxylic acids, enabling their structure determination (Smith & Wermuth, 2010*a*, 2010*b*). The structure of the 2:1 INIPA salt of biphenyl-4,4'-disulfonic acid has also been reported (Smith *et al.*, 2010), and all reported compounds, prepared in aqueous ethanolic solution, have been anhydrous. No structures with aliphatic acids have previously been reported. However, our reaction of isonipecotamide with acetic acid in aqueous methanolic solution gave the title compound, the hydrate C_6_H_13_N_2_O^+^ C_2_H_3_O_2_^-^. H_2_O, (I) and the structure is reported here.

With (I) (Fig. 1) the amide H atoms of the cations form centrosymetric cyclic hydrogen-bonding associations which incorporate two water molecules [graph set *R*^2^_4_(8) (Etter *et al.*, 1990)], These are conjoint with cyclic water-bridged amide–amide associations [*R*_4_^4^(12)] and larger *R*_4_^4^(20) associations also involving the water molecule and the acetate anions (Table 1). These acetate groups bridge the cations through the piperidinium H donor atoms, giving an overall three-dimensional framework structure (Fig. 2).

### Experimental

The title compound was synthesized by heating together under reflux for 10 minutes, 1 mmol quantities of 4-carbamoylpiperidine (isonipecotamide) and acetic acid in 50 ml of 80% methanol–water. After concentration to *ca* 30 ml, partial room temperature evaporation of the hot-filtered solution gave colourless plates of (I) (m.p. 409 K) from which a specimen was cleaved for the X-ray analysis.

### Refinement

Hydrogen atoms involved in hydrogen-bonding interactions were located by difference methods and their positional and isotropic displacement parameters were refined. The N–H bond distance range is 0.899 (17)–0.949 (18) Å and the water O–H distances are 0.82 (2) and 0.92 (2) Å. Other H-atoms were included in the refinement at calculated positions [C–H = 0.96–0.97 Å and with *U*_iso_(H) = 1.2*U*_eq_(C), using a riding-model approximation.

### Crystal data

**Table:**

C_6_H_13_N_2_O^+^·C_2_H_3_O_2_^−^·H_2_O	*Z* = 2
*M**_r_* = 206.24	*F*(000) = 224
Triclinic, *P*1	*D*_x_ = 1.291 Mg m^−^^3^
Hall symbol: -P 1	Melting point: 409 K
*a* = 5.8219 (2) Å	Mo *K*α radiation, λ = 0.71073 Å
*b* = 7.7675 (3) Å	Cell parameters from 3330 reflections
*c* = 12.4022 (5) Å	θ = 3.4–28.8°
α = 81.088 (4)°	µ = 0.10 mm^−^^1^
β = 78.763 (4)°	*T* = 200 K
γ = 76.202 (4)°	Plate, colourless
*V* = 530.75 (4) Å^3^	0.40 × 0.35 × 0.15 mm

### Data collection

**Table:**

Oxford Diffraction Gemini-S Ultra CCD-detector diffractometer	2087 independent reflections
Radiation source: Enhance (Mo) X-ray source	1602 reflections with *I* > 2σ(*I*)
graphite	*R*_int_ = 0.021
Detector resolution: 16.977 pixels mm^-1^	θ_max_ = 26.0°, θ_min_ = 3.4°
ω scans	*h* = −7→7
Absorption correction: multi-scan (*CrysAlis PRO*; Oxford Diffraction, 2010)	*k* = −9→9
*T*_min_ = 0.962, *T*_max_ = 0.980	*l* = −15→15
6385 measured reflections	

### Refinement

**Table:**

Refinement on *F*^2^	Primary atom site location: structure-invariant direct methods
Least-squares matrix: full	Secondary atom site location: difference Fourier map
*R*[*F*^2^ > 2σ(*F*^2^)] = 0.035	Hydrogen site location: inferred from neighbouring sites
*wR*(*F*^2^) = 0.105	H atoms treated by a mixture of independent and constrained refinement
*S* = 0.93	*w* = 1/[σ^2^(*F*_o_^2^) + (0.0733*P*)^2^ + 0.0214*P*] where *P* = (*F*_o_^2^ + 2*F*_c_^2^)/3
2087 reflections	(Δ/σ)_max_ < 0.001
151 parameters	Δρ_max_ = 0.20 e Å^−^^3^
0 restraints	Δρ_min_ = −0.19 e Å^−^^3^

### Special details

**Table:**

Geometry. Bond distances, angles *etc*. have been calculated using the rounded fractional coordinates. All su's are estimated from the variances of the (full) variance-covariance matrix. The cell e.s.d.'s are taken into account in the estimation of distances, angles and torsion angles
Refinement. Refinement of *F*^2^ against ALL reflections. The weighted *R*-factor *wR* and goodness of fit *S* are based on *F*^2^, conventional *R*-factors *R* are based on *F*, with *F* set to zero for negative *F*^2^. The threshold expression of *F*^2^ > σ(*F*^2^) is used only for calculating *R*-factors(gt) *etc*. and is not relevant to the choice of reflections for refinement. *R*-factors based on *F*^2^ are statistically about twice as large as those based on *F*, and *R*- factors based on ALL data will be even larger.

### Fractional atomic coordinates and isotropic or equivalent isotropic displacement parameters (Å^2^)

**Table:**

	*x*	*y*	*z*	*U*_iso_*/*U*_eq_	
O41A	0.48609 (16)	0.38864 (13)	0.35243 (9)	0.0344 (3)	
N1A	0.9204 (2)	0.82745 (15)	0.19803 (10)	0.0274 (4)	
N41A	0.7954 (2)	0.21185 (16)	0.42814 (10)	0.0297 (4)	
C2A	0.9917 (3)	0.67001 (19)	0.13552 (11)	0.0290 (4)	
C3A	0.8415 (2)	0.53343 (18)	0.18703 (11)	0.0264 (4)	
C4A	0.8642 (2)	0.47820 (17)	0.30875 (11)	0.0223 (4)	
C5A	0.8010 (3)	0.64377 (18)	0.37045 (11)	0.0283 (4)	
C6A	0.9525 (3)	0.77742 (19)	0.31557 (12)	0.0319 (5)	
C41A	0.6994 (2)	0.35378 (17)	0.36412 (11)	0.0236 (4)	
O11	1.43436 (17)	−0.04485 (13)	0.19784 (8)	0.0320 (3)	
O12	1.14721 (18)	0.09626 (14)	0.10165 (9)	0.0395 (4)	
C1	1.3462 (2)	0.08082 (17)	0.12972 (11)	0.0246 (4)	
C2	1.4884 (3)	0.2219 (2)	0.08190 (13)	0.0375 (5)	
O1W	1.31002 (18)	0.08400 (15)	0.40367 (9)	0.0314 (3)	
H4A	1.03030	0.41690	0.31380	0.0270*	
H11A	1.005 (3)	0.917 (2)	0.1664 (15)	0.050 (5)*	
H12A	0.757 (3)	0.882 (2)	0.1956 (14)	0.043 (5)*	
H21A	1.16000	0.61660	0.13610	0.0350*	
H22A	0.96950	0.70700	0.05920	0.0350*	
H31A	0.89420	0.42910	0.14720	0.0320*	
H32A	0.67480	0.58390	0.18070	0.0320*	
H41A	0.958 (3)	0.178 (2)	0.4274 (13)	0.036 (4)*	
H42A	0.701 (3)	0.138 (2)	0.4646 (14)	0.045 (5)*	
H51A	0.63270	0.69920	0.37160	0.0340*	
H52A	0.82710	0.60850	0.44640	0.0340*	
H61A	0.90520	0.88300	0.35420	0.0380*	
H62A	1.12010	0.72560	0.31940	0.0380*	
H21	1.42940	0.28690	0.01690	0.0450*	
H22	1.65450	0.16610	0.06270	0.0450*	
H23	1.47180	0.30250	0.13570	0.0450*	
H11W	1.355 (4)	0.031 (3)	0.3388 (19)	0.069 (7)*	
H12W	1.368 (4)	0.175 (3)	0.3891 (17)	0.062 (6)*	

### Atomic displacement parameters (Å^2^)

**Table:**

	*U*^11^	*U*^22^	*U*^33^	*U*^12^	*U*^13^	*U*^23^
O41A	0.0236 (5)	0.0304 (6)	0.0475 (6)	−0.0104 (4)	−0.0061 (4)	0.0086 (5)
N1A	0.0228 (6)	0.0200 (6)	0.0383 (7)	−0.0088 (5)	−0.0057 (5)	0.0076 (5)
N41A	0.0279 (7)	0.0236 (6)	0.0349 (7)	−0.0100 (5)	−0.0026 (5)	0.0083 (5)
C2A	0.0309 (7)	0.0300 (8)	0.0250 (7)	−0.0108 (6)	−0.0021 (6)	0.0039 (6)
C3A	0.0310 (7)	0.0259 (7)	0.0235 (7)	−0.0116 (6)	−0.0025 (5)	−0.0007 (6)
C4A	0.0192 (6)	0.0198 (6)	0.0269 (7)	−0.0060 (5)	−0.0038 (5)	0.0029 (5)
C5A	0.0345 (8)	0.0271 (7)	0.0247 (7)	−0.0124 (6)	−0.0020 (6)	−0.0016 (6)
C6A	0.0391 (8)	0.0270 (8)	0.0336 (8)	−0.0155 (6)	−0.0057 (6)	−0.0032 (6)
C41A	0.0239 (7)	0.0202 (7)	0.0256 (7)	−0.0065 (5)	−0.0007 (5)	−0.0008 (6)
O11	0.0274 (5)	0.0289 (5)	0.0362 (6)	−0.0050 (4)	−0.0060 (4)	0.0061 (5)
O12	0.0333 (6)	0.0339 (6)	0.0535 (7)	−0.0167 (5)	−0.0175 (5)	0.0165 (5)
C1	0.0250 (7)	0.0227 (7)	0.0257 (7)	−0.0074 (5)	−0.0012 (5)	−0.0015 (6)
C2	0.0368 (8)	0.0335 (8)	0.0450 (9)	−0.0182 (7)	−0.0080 (7)	0.0054 (7)
O1W	0.0325 (6)	0.0304 (6)	0.0324 (6)	−0.0149 (5)	−0.0066 (4)	0.0073 (5)

### Geometric parameters (Å, °)

**Table:**

O41A—C41A	1.2386 (16)	C4A—C41A	1.5186 (18)
O11—C1	1.2667 (16)	C5A—C6A	1.518 (2)
O12—C1	1.2473 (17)	C2A—H21A	0.9700
O1W—H12W	0.84 (2)	C2A—H22A	0.9700
O1W—H11W	0.92 (2)	C3A—H32A	0.9700
N1A—C6A	1.4856 (19)	C3A—H31A	0.9700
N1A—C2A	1.4816 (18)	C4A—H4A	0.9800
N41A—C41A	1.3289 (18)	C5A—H51A	0.9700
N1A—H12A	0.949 (18)	C5A—H52A	0.9700
N1A—H11A	0.940 (17)	C6A—H62A	0.9700
N41A—H41A	0.919 (18)	C6A—H61A	0.9700
N41A—H42A	0.899 (17)	C1—C2	1.509 (2)
C2A—C3A	1.521 (2)	C2—H23	0.9600
C3A—C4A	1.5266 (19)	C2—H21	0.9600
C4A—C5A	1.5311 (19)	C2—H22	0.9600
			
H11W—O1W—H12W	104 (2)	C2A—C3A—H32A	109.00
C2A—N1A—C6A	111.60 (11)	C4A—C3A—H32A	109.00
H11A—N1A—H12A	104.9 (14)	H31A—C3A—H32A	108.00
C6A—N1A—H11A	109.2 (11)	C4A—C3A—H31A	109.00
C2A—N1A—H11A	112.5 (10)	C5A—C4A—H4A	109.00
C2A—N1A—H12A	109.7 (10)	C41A—C4A—H4A	109.00
C6A—N1A—H12A	108.8 (10)	C3A—C4A—H4A	109.00
C41A—N41A—H42A	118.4 (11)	C4A—C5A—H51A	109.00
C41A—N41A—H41A	121.8 (10)	C6A—C5A—H51A	109.00
H41A—N41A—H42A	119.1 (15)	C6A—C5A—H52A	109.00
N1A—C2A—C3A	110.23 (12)	C4A—C5A—H52A	109.00
C2A—C3A—C4A	110.91 (11)	H51A—C5A—H52A	108.00
C3A—C4A—C41A	111.67 (10)	N1A—C6A—H62A	110.00
C5A—C4A—C41A	108.66 (11)	C5A—C6A—H61A	110.00
C3A—C4A—C5A	109.87 (11)	C5A—C6A—H62A	110.00
C4A—C5A—C6A	111.11 (12)	H61A—C6A—H62A	108.00
N1A—C6A—C5A	109.84 (13)	N1A—C6A—H61A	110.00
O41A—C41A—C4A	120.90 (12)	O11—C1—O12	123.79 (12)
O41A—C41A—N41A	122.94 (12)	O11—C1—C2	117.89 (12)
N41A—C41A—C4A	116.11 (11)	O12—C1—C2	118.31 (12)
N1A—C2A—H21A	110.00	C1—C2—H22	109.00
C3A—C2A—H21A	110.00	C1—C2—H23	109.00
C3A—C2A—H22A	110.00	C1—C2—H21	109.00
H21A—C2A—H22A	108.00	H21—C2—H23	109.00
N1A—C2A—H22A	110.00	H22—C2—H23	109.00
C2A—C3A—H31A	109.00	H21—C2—H22	109.00
			
C6A—N1A—C2A—C3A	59.57 (16)	C41A—C4A—C5A—C6A	−177.38 (12)
C2A—N1A—C6A—C5A	−59.60 (16)	C3A—C4A—C41A—O41A	−47.52 (17)
N1A—C2A—C3A—C4A	−56.87 (15)	C3A—C4A—C41A—N41A	135.05 (12)
C2A—C3A—C4A—C5A	54.57 (15)	C5A—C4A—C41A—O41A	73.82 (16)
C2A—C3A—C4A—C41A	175.21 (11)	C5A—C4A—C41A—N41A	−103.61 (14)
C3A—C4A—C5A—C6A	−54.94 (16)	C4A—C5A—C6A—N1A	57.10 (16)

### Hydrogen-bond geometry (Å, °)

**Table:**

*D*—H···*A*	*D*—H	H···*A*	*D*···*A*	*D*—H···*A*
N1A—H11A···O12^i^	0.940 (17)	1.793 (17)	2.7311 (16)	175.8 (17)
N1A—H12A···O11^ii^	0.949 (18)	1.824 (18)	2.7666 (16)	171.8 (14)
N41A—H41A···O1W	0.919 (18)	1.984 (18)	2.8939 (17)	170.2 (15)
N41A—H42A···O1W^iii^	0.899 (17)	2.188 (16)	2.9491 (16)	142.1 (15)
O1W—H11W···O11	0.92 (2)	1.87 (2)	2.7871 (15)	172 (2)
O1W—H12W···O41A^iv^	0.84 (2)	1.90 (2)	2.7370 (15)	177 (2)
C2A—H22A···O12^v^	0.97	2.42	3.3428 (18)	158

Symmetry codes: (i) *x*, *y*+1, *z*; (ii) *x*−1, *y*+1, *z*; (iii) −*x*+2, −*y*, −*z*+1; (iv) *x*+1, *y*, *z*; (v) −*x*+2, −*y*+1, −*z*.
